# Variant surface antigens in cerebral malaria: distinct from others and similar to each other?

**DOI:** 10.1186/1475-2875-9-S2-O2

**Published:** 2010-10-20

**Authors:** Agnès Aubouy, Nabila Kheliouen, Nicaise Tuikue-Ndam, Firmine Viwami, Francis Lalya, Else C Eboumbou Moukoko, Christophe Rogier, Philippe Deloron

**Affiliations:** 1 Institut de Recherche pour le Développement(IRD) UMR216, Paris, 75006 France; 2 Université Paris Descartes, Faculté de Pharmacie, Paris, 75270, France; 3 Paediatric Department, Centre National Hospitalo-Universitaire (CNHU), Cotonou, Benin; 4 Unité de recherche en biologie et épidémiologie parasitaires, Institut de Recherche Biomédicale des Armées, UMR6236, Marseille, France; 5 Département des Sciences Biologiques, Faculté de Médecine et des Sciences Pharmaceutiques, Université de Douala, BP2701, Douala, Cameroun

## 

Immunological protection against *Plasmodium falciparum* blood stages is mainly antibody mediated [1,2]. Variant surface antigens (VSA) expressed on the surface of *P. falciparum*-infected red blood cells constitute a key for parasite sequestration and immune evasion [3]. In distinct malaria clinical presentations, as placental malaria, specific antibody response against VSA provides protection [4].

In the current study, we investigated in distinct clinical groups of malaria patients, the antibody response specifically directed against VSA expressed by parasites isolated from a given clinical presentation, and particularly isolates obtained from cerebral malaria (CM) patients. Plasma and isolates were obtained from four groups of Beninese subjects: healthy adults (HA, n = 34), patients presenting uncomplicated malaria (UM, n = 62), cerebral malaria (CM, n = 41), or pregnancy-associated malaria (PAM, n = 24). Isolates were tested for their clonality by *msp1* and *msp2* genotyping. The reactivity of plasma samples from each clinical group was measured by flow cytometry against parasites isolated from individuals from each clinical group.

The levels of clonality were similar in isolates from all clinical origins. In healthy adults and children presenting UM, VSA_UM_ antibody levels were higher than VSA_CM_ antibody levels (Figure 1). In both PAM plasma groups (primigravidae and multigravidae), antibody levels against the three types of isolates were similar. One month after infection the level of anti-VSA antibodies able to recognize heterologous VSA_CM_ variants was increased in CM patients. In UM patients, antibody levels directed against heterologous VSA_UM_ were similar during the infection and one month later (Figure 2).

**Figure 1:**
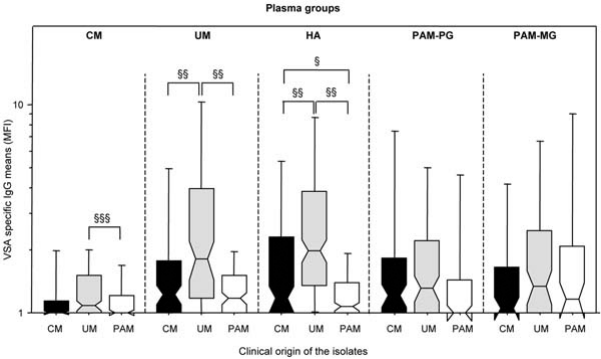
Relative levels of VSA specific IgG to heterologous *P.falciparum* isolates according to the clinical origin of the *P. falciparum* isolates, and to the plasma group. PG: primigravidae, MG: multigravidae. Errors bars indicate standard errors. Groups were compared all together by ANCOVA (*** P <.0005, * P <.05, ns. non significant), and two factor ANOVA (^§§§^ P < .0005, ^§§^ P < .005, ^§^ P < .05).

**Figure 2:**
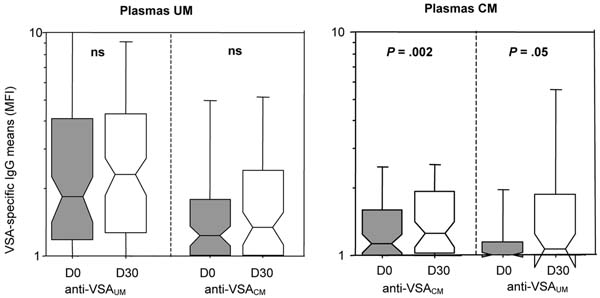
Acquisition of VSA-specific IgG during the month following *P. falciparum* infection in plasmas samples from UM (n = 121) and CM (n = 56) patients. UM (A, B) or CM (C, D) plasma samples were tested against *P. falciparum* isolates from UM patients (A, D) or from CM patients (B, C). Centerlines indicate medians, boxes indicate the 25th and 75th percentiles of data points, bars indicate the 10th and 90th percentiles and circles are outliers. Differences are derived from the Wilcoxon rank test for paired comparisons, ns: non significant.

The existence of shared VSA_CM_ epitopes was shown but does not necessarily involve prevalent epitopes. Prevalence is more probably due to a fine balance between transmission intensity, antibody repertoire and environmental factors.

## References

[B1] Cohen S McGregor IA Carrington S Gamma-globulin and acquired immunity to human malaria Nature 1961 192 733 7 10.1038/192733a0 13880318

[B2] Bouharoun-Tayoun H Attanath P Sabchareon A Chongsuphajaisiddhi T Druilhe P Antibodies that protect humans against *Plasmodium falciparum* blood stages do not on their own inhibit parasite growth and invasion in vitro, but act in cooperation with monocytes J Exp Med 1990 172 1633 41 10.1084/jem.172.6.1633 2258697 PMC2188756

[B3] Bull PC Lowe BS Kortok M Molyneux CS Newbold CI Marsh K Parasite antigens on the infected red cell are targets for naturally acquired immunity to malaria Nat Med 1998 4 358 60 10.1038/nm0398-358 9500614 PMC3836255

[B4] Brabin BJ An analysis of malaria in pregnancy in Africa Bull World Health Organ 1983 61 1005 16 6370484 PMC2536236

